# Molecular Characterization of Novel Family IV and VIII Esterases from a Compost Metagenomic Library

**DOI:** 10.3390/microorganisms9081614

**Published:** 2021-07-29

**Authors:** Jong-Eun Park, Geum-Seok Jeong, Hyun-Woo Lee, Hoon Kim

**Affiliations:** Department of Pharmacy, Research Institute of Life Pharmaceutical Sciences, Sunchon National University, Suncheon 57922, Korea; 1200113@s.scnu.ac.kr (J.-E.P.); 1200058@s.scnu.ac.kr (G.-S.J.); hwlee@rophibio.com (H.-W.L.)

**Keywords:** compost metagenomic library, family IV esterase, family VIII esterase, glyceryl tributyrate hydrolysis, oligomeric native form, organic solvent stability, broad substrate specificity

## Abstract

Two novel esterase genes, *est*8L and *est*13L, were isolated and identified from a compost metagenomic library. The encoded Est8L and Est13L had molecular masses of 33,181 and 44,913 Da consisting of 314 and 411 amino acids, respectively, without signal peptides. Est8L showed the highest identity (32.9%) to a hyper-thermophilic carboxylesterase AFEST from *Archaeoglobus* *fulgidus* compared to other esterases reported and was classified to be a novel member of family IV esterases with conserved regions such as HGGG, DY, GXSXG, DPL, and GXIH. Est13L showed the highest identity (98.5%) to the family VIII esterase Est7K from the metagenome library. Est8L and Est13L had the highest activities for *p*-nitrophenyl butyrate (C4) and *p*-nitrophenyl caproate (C6), respectively, and Est13L showed a broad substrate specificity for *p*-nitrophenyl substrates. Est8L and Est13L effectively hydrolyzed glyceryl tributyrate. The optimum temperatures for activities of Est8L and Est13L were identical (40 °C), and the optimum pH values were 9.0 and 10.0, respectively. Est13L showed higher thermostability than Est8L. Sephacryl S-200 HR chromatography showed that the native form of Est8L was a dimer. Interestingly, Est13L was found to be a tetramer, contrary to other family VIII esterases reported. Est8L was inhibited by 30% isopropanol, methanol, and acetonitrile; however, Est13L was activated to 182.9% and 356.1%, respectively, by 30% isopropanol and methanol. Est8L showed enantioselectivity for the *S*-form, but Est13L showed no enantioselectivity. These results show that intracellular Est8L and/or Est13L are oligomeric in terms of native forms and can be used for pharmaceutical and industrial applications with organic solvents under alkaline conditions.

## 1. Introduction

Lipolytic enzymes, such as esterase (EC 3.1.1.1) and lipase (EC 3.1.1.3), hydrolyze the ester bond to carboxylic acid and alcohol. Esterases hydrolyze ester bonds in short chains with <10 carbons, whereas lipase hydrolyzes ester bonds in long chains with >10 carbons [1]. The application of esterases and lipases has been widely researched for chemical reactions and for application in the pulp and paper, cosmetics, and pharmaceutical industries [2,3]. Especially, esterases are applied for the improvement of the lipophilicity for ester prodrugs to increase cell wall penetration [4]. Bacterial esterases were first classified into eight families based on their primary amino acid sequences [1], and recently other families have been reported up to the 19th family [5].

The family IV esterase, which is called hormone-sensitive lipase (HSL), has a catalytic triad consisting of serine (S), aspartic acid (D), and histidine (H). The serine residue in the catalytic triad is placed in the conserved GXSXG motif [6]. The bacterial HSL (bHSL) is a member of the α/β hydrolase family, which have similar structures, i.e., β-sheets covered by α-helixes [7]. The bHSL is divided into two domains: cap and catalytic domains. The catalytic domain forms α-helix and β-sheets with catalytic residues, and the cap domain is located on the upper side of the catalytic residues, which is associated with substrate recognition [8].

Three enzymes showing similarity to C-β-lactamase were first classified as family VIII members. The enzymes showed a typical conserved region SxxK. Their catalytic residues include S and lysine (K) in the SxxK motif and tyrosine (Y), unlike other families containing S, D, and H [9].

Family IV and VIII esterases have been practically interested in the usage of ester synthesis, transesterification reactions, and hydrolysis of antibiotics [10,11].

Numerous bacterial esterases have been mainly isolated from metagenomic libraries. The metagenome is the collected genome of whole microorganisms in an environment without culture [12]. The DNA sample is directly extracted from sediments [13] and is called environmental DNA (eDNA) [14,15], serving extremely diverse genes. Numerous recent studies have focused on metagenomes from various environments, such as Antarctic soil [16], deep-sea [17], oil-polluted mudflat [18], volcano soil [19], and plant wasted water [8].

Compost contains diverse microorganisms producing various hydrolytic enzymes, including esterases and lipases [20]. Our library was constructed using compost metagenome, and 19 esterase-positive fosmid clones were obtained; then, the positive clones were mixed together, and extracted DNA was subcloned using cloning vector to obtain 18 positive subclones [21], two esterases of which, Est2K and Est7K, were characterized [21,22]. In this study, we identified two novel genes, *est*8L and *est*13L, from the library, and the encoded Est8L and Est13L esterases were characterized.

## 2. Materials and Methods

### 2.1. Materials

*p*-Nitrophenyl (pNP) esters from pNP-acetate (C2) to pNP-palmitate (C16), glyceryl esters (glyceryl tributyrate, glyceryl trioctanoate, and glyceryl trioleate), oils (fish oil and olive oil), acetylthiocholine iodide (ATCI), enantiomers [(*R*)- and (*S*)-methyl-3-hydroxy-2-methyl-propionate], *S*-butyrylthiocholine iodide (BTCI), and 5,5’-dithiobis(2-nitrobenzoic acid) (DTNB) were purchased from Sigma-Aldrich (St. Louis, MO, USA). Columns for purification such as HiTrap Q HP (5 mL), *t*-butyl hydrophobic interaction chromatography (HIC) (1 mL), HiTrap capto MMC (1 mL), and HiPrep 16/60 Sephacryl S-200 HR were purchased from GE Healthcare (Uppsala, Sweden).

### 2.2. Two Esterase-Positive Clones from the Compost Metagenome Library

The compost metagenome was obtained from Yonghyun Nonghyub Compost Factory (Sachon, Korea), and completely fermented compost was used for metagenomic DNA extraction [21]. A metagenomic library was constructed using fosmid from the compost previously, and 19 esterase-positive fosmid clones were obtained [21]. By mixing, 18 positive subclones were obtained, and nine different lipolytic genes were identified by sequencing; two of them were selected based on the halo and the lowest similarities, and their characteristics were reported [21,22]. We selected two esterase-positive subclones further and their genes *est*8L and *est*13L. The transformants were incubated on LB agar plates containing 1% glyceryl tributyrate for 12 h at 25 °C.

### 2.3. Sequence Analysis and Phylogenetic Tree

Recombinant plasmids were isolated from the clones, and DNA sequences were determined by the dideoxy method (http://www.solgent.com/english/sub03030101, accessed on 25 July 2021) at Solgent (Daejeon, Korea). Amino acid sequences and conserved regions were analyzed by BLASTp of NCBI (http://www.ncbi.nlm.nih.gov, accessed on 12 April 2021). Putative signal peptides were predicted by SignalP 5.0 in CBS (http://www.cbs.dtu.dk/services/SignalP/, accessed on 12 April 2021), and molecular mass and theoretical pI were calculated using the ExPASy ProtParam tool (http://web.expasy.org/protparam, accessed on 11 April 2021). Multiple sequence alignments were analyzed and performed using the Clustal W method in DNA/MAN (Lynnon Biosoft, version 4.11, Quebec, Canada). Phylogenetic trees were constructed by MEGA version X [23] using the maximum likelihood method.

### 2.4. Preparation of Crude Enzymes

Esterase-positive clones were inoculated in 200 mL LB broth and incubated for 15 h at 200 rpm and 37 °C. After incubation, the medium was centrifuged for 15 min at 6000× *g* and 4 °C. The pellet was washed two times with 20 mL of 20 mM Tris-HCl (pH 8.0) and centrifuged for 5 min at 6000× *g* and 4 °C. The pellet was resuspended in 5 mL of the same buffer and sonicated three times under an amplitude of 38% and pulse on for 1 sec and pulse off for 1 sec for 1 min using a microtip equipped on a sonicator (VCX500, Sonics & Materials, Newtown, CT, USA). After centrifugation for 15 min at 6000× *g* and 4 °C, the supernatant was collected as a crude enzyme.

### 2.5. Purification of the Enzymes and Activity Staining

The crude enzymes were separated further using a Biologic LP System (Bio-Rad). HiTrap Q anion exchange chromatography was performed using 20 mM Tris-HCl (pH 8.0) as a low buffer and 20 mM Tris-HCl (pH 8.0) containing 1 M NaCl as a high buffer at a flow rate of 1.0 mL/min for 90 min. The HiTrap t-butyl HIC was employed as a second column using 50 mM sodium phosphate (pH 7.0) buffer containing 1.5 M (NH_4_)_2_SO_4_ as a high buffer and 50 mM sodium phosphate (pH 7.0) buffer as a low buffer at a flow rate of 1.0 mL/min for 60 min. The HiTrap capto MMC was performed for further purification using 25 mM sodium acetate (pH 4.5) buffer as a low buffer and 50 mM sodium phosphate (pH 7.0) buffer including 1 M NaCl as a high buffer at a flow rate of 0.5 mL/min for 2 h. Size exclusion chromatography was performed using Sephacryl S-200 with 50 mM sodium phosphate (pH 7.0) containing 0.15 M NaCl at a flow rate of 0.5 mL/min for 240 min. Molecular mass standards were used with β-amylase, bovine serum albumin (BSA), and trypsinogen (200, 66.4, and 24.0 kDa, respectively).

To confirm the activity and molecular mass of each cloned enzyme at the same time, HiTrap Q pools were separated by native polyacrylamide gel electrophoresis (PAGE) without sodium dodecyl sulfate (SDS) and heat treatment of the sample, and the gel was analyzed by activity staining. The native gel was soaked with 50 mM Tris-HCl (pH 8.0) for 30 min, tightly overlapped to a 3% agar strip containing 1.5% glyceryl tributyrate, wrapped, and incubated for 30 min at 40 °C until a clear band was visible. The region of the gel corresponding to the clear band of the strip was sliced, chopped, and eluted in 20 mM Tris-HCl buffer overnight at 4 °C. After centrifugation for 5 min at 10,000× *g* and 4 °C, the supernatant was collected and used as a purified enzyme. To verify the purity, SDS-PAGE was performed by loading the samples in 11.5% acrylamide gel [24]; then, proteins in the gel were stained with silver. Protein concentration was determined by the Bradford assay [25].

### 2.6. Enzyme Assays

The esterase activity was calculated as the amount of *p*-nitrophenol, which is the product of the reaction between esterase and *p*-nitrophenyl ester. The enzyme was added into 50 mM Tris-HCl buffer (pH 8.0) containing 1 mM pNPB. The absorbances of the reaction mixtures were measured continuously for 2 min at 400 nm and 25 °C as a standard assay using the kinetic mode of a spectrophotometer (Optizen, K-Lab, Daejon, Korea). One unit of the enzyme activity was the enzyme amount producing 1 μmol of *p*-nitrophenol per minute using a molar extinction coefficient of 16,400/M/cm at pH 8.0. Acetylcholinesterase and butyrylcholinesterase activities were measured using ATCI and BTCI, respectively, as described previously [26]. Enzymes reacted with 0.5 mM substrate and 0.5 mM DTNB in 100 mM sodium phosphate (pH 7.5), and the reaction was monitored continuously for 10 min at 412 nm and 25 °C in the kinetic mode.

### 2.7. Characterization of Enzymes

The optimum temperature was identified using the standard assay method at 20, 30, 40, 50, 60, and 70 °C. The optimum pH was determined using 50 mM universal buffer (boric acid/citric acid/trisodium orthophosphate) in the range of pH 6.0 and 11.0, and molecular extinction of each pH was used, as described previously [18]. The heat stability of the purified enzyme was analyzed by preincubating at 30, 40, 50, and 60 °C for 1 h at designated times.

Substrate specificity was investigated using 1 mM *p*-NP esters (C2 ~ C16). Five different concentrations of substrate, i.e., pNPB (C4), were used for the kinetic study, and Lineweaver–Burk plots were constructed to calculate the K_m_ and V_max_.

The ion effect on enzyme activity was investigated by adding Na^+^, K^+^, Mg^2+^, Ca^2+^, Ba^2+^, Mn^2+^, Fe^2+^, Co^2+^, Cu^2+^, and Zn^2+^ ions at 2 and 5 mM of concentrations. The organic solvent stability of the enzyme was observed at 5% or 30%, such as methanol, isopropanol, and acetonitrile. The detergent stability was analyzed at 1% SDS or Triton-X-100.

The lipid hydrolysis activity of the enzyme was confirmed using a pH shift assay [27] using 1% oils (fish oil and olive oil) or glyceryl triesters (glyceryl tributyrate, glyceryl trioctanoate, and glyceryl trioleate) and 0.1% phenol red in 20 mM Tris-HCl (pH 8.0). Absorbance was measured using a spectrophotometer in the kinetic mode at every 5 min for 60 min at 560 nm and 25 °C, and the amount of remaining substrate was determined. To confirm the enantioselectivity of the enzyme, 1% (*R*)- or (*S*)-methyl-3-hydroxy-2-methyl-propionate was used as a substrate [18].

### 2.8. Accession Numbers of the Est8L and Est13L

The sequences of *est*8L and *est*13Lwere deposited under accession numbers MZ484407 and MZ484408 at GenBank, respectively.

## 3. Results

### 3.1. Sequence Analyses and Multiple Alignments of Est8L and Est13L

**Figure 1:**
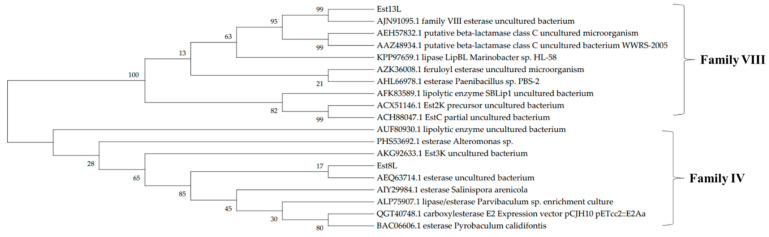
A phylogenetic tree of Est8L and Est13L using the maximum likelihood method. The number at the node shows bootstrap percentages of 1000 replicates. The accession numbers were from GenBank.

The positive clones YH-E8 and YH-E13 were selected in this study based on their halo sizes. The insert DNA of YH-E8 was 3202 bp in length, and an open reading frame (ORF) of 945 bp coding an esterase was identified and named *est*8L. The insert DNA of YH-E13 was 3871 bp long, and an ORF of an esterase consisting of 1236 bp was identified and named *est*13L. The encoded proteins Est8L and Est13L had molecular weights of 33,181 and 44,913 Da, respectively, were consisted of 314 and 411 amino acid residues, respectively; their theoretical pI values were 4.66 and 6.34, respectively. Est8L and Est13L had no signal peptides, as observed by SignalP-5.0 analysis. Analysis of the phylogenetic tree predicted that Est8L was a novel member of family IV esterases, and Est13L was a novel member of family VIII esterases (Figure 1). A BLASTp search showed that Est8L has the highest identity (100%) for an alpha/beta hydrolase from *Sphingorhabdus* sp. (GenBank MBF6602187) in annotated sequences. However, Est8L showed the highest identity (32.9%) to a hyper-thermophilic carboxylesterase AFEST from *Archaeoglobus fulgidus* compared to other esterases enzymatically reported to date. Est13L had the highest identity (98.5%) for the family VIII esterase Est7K from an uncultured bacterium (AJN91095).

**Figure 2:**
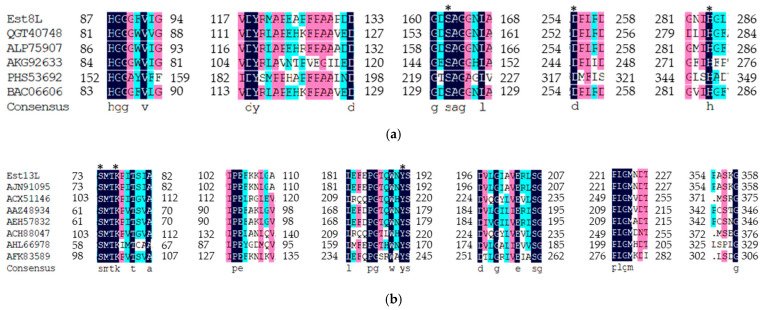
Multiple sequence alignments of Est8L (**a**) and Est13L (**b**) using the Clustal W method in DNA/MAN. The asterisks represent the catalytic triads. Family IV esterases: QGT40748, carboxyl esterase E2 (expression vector pCJH10_pETcc2::E2Aa); ALP75907, lipase/esterase (*Parvibaculum* sp. enrichment culture); AKG92633, Est3K (uncultured bacterium); PHS53692, esterase (*Altermonas* sp.); esterase (*Pyrobaculum calidifontis*). Family VIII esterases: AJN91095, family VIII esterase (Est7K) (uncultured bacterium); ACX51146, Est2K precursor (uncultured bacterium); AAZ48934, putative beta-lactamase class C (uncultured bacterium WWRS-2005); AEH57832, putative beta-lactamase class C (uncultured microorganism); ACH88047, EstC, partial (uncultured bacterium); AHL66978, esterase (*Paenibacillus* sp. PBS-2); lipolytic enzyme SBLip1 (uncultured bacterium).

In multiple sequence alignment (MSA) of Est8L with other family IV lipolytic enzymes, some conserved regions such as HGGG (corresponding to 87–89 amino acid residues), DY (118–119), GXSAG (160–164), DPL (254–256), and GXIH (281–284) were confirmed, and the underlined serine (S), aspartic acid (D), and histidine (H) were predicted to form a catalytic triad of alpha/beta hydrolase (Figure 2a). From the MSA of Est13L with other family VIII lipolytic enzymes, numerous conserved regions such as SMTK (73–76), IPE (102–104), LXXXPGXXWXYS (181–192), DXXGXXXEXXSG (196–207), and PLGM (221–224) were identified. Similar to most of the family VIII esterases, Est13L had XXSXG (354–358) instead of GXSXG, which is commonly observed in esterase. However, in family VIII esterases, the serine residue of the catalytic triad is derived from the SMTK motif, not XXSXG (Figure 2).

### 3.2. Purification of Est8L and Est13L

**Figure 3:**
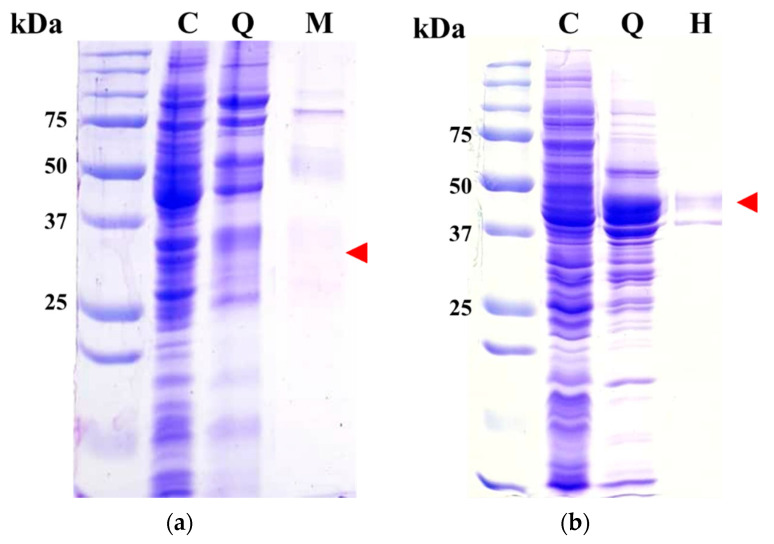
Gels of purified Est8L (**a**) and Est13L (**b**) after SDS-PAGE and staining with Coomassie blue. Red arrows in (**a**) and (**b**) indicate the predicted positions of Est8L and Est13L, respectively. C, crude extract; Q, HiTrap Q fraction; M, HiTrap capto MMC fraction; H, *t*-butyl HIC fraction.

Each enzyme of Est8L and Est13L was purified using HiTrap Q as the first column. Both enzymes were bound to the resin and eluted through a gradient step (Supplementary Figures S1a and S2a). Next, a t-butyl HIC column was employed as a second column; however, recovery of Est8L activity was too low to be detectable, whereas Est13L was successfully purified. Therefore, HiTrap capto MMC and HIC columns were used as the second columns for Est8L and Est13L, respectively (Supplementary Figures S1b and S2b). In SDS-PAGE, purified Est8L was observed with smear bands at the predicted position of the gel, i.e., corresponding to ~33 kDa (Figure 3a), probably because of the internal proteolytic cleavage of foreign proteins as in other cases [28,29,30,31,32]. Est13L appeared as a major band at the predicted position, corresponding to ~45 kDa (Figure 3b).

Through this procedure, the purification fold and yield of Est8L were 11.9% and 12.1%, respectively, and those of Est13L were 40.3% and 43.2%, respectively (Table 1).

**Figure 4:**
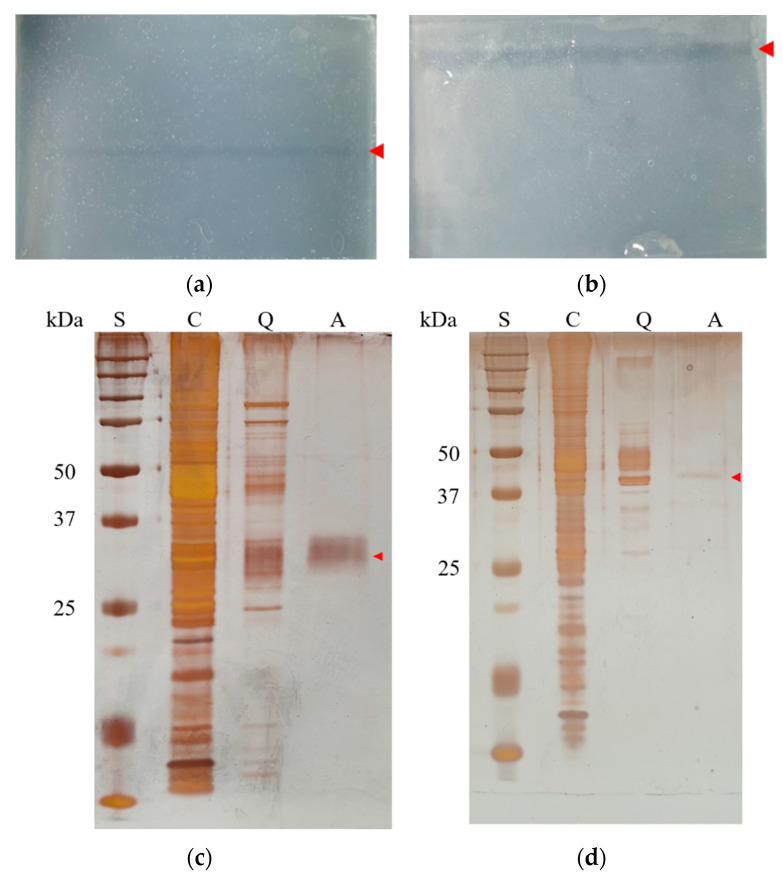
Activity staining of Est8L (**a**) and Est13L (**b**) onto the agar strips after native-PAGE and analysis of recovered Est8L (**c**) and Est13L (**d**) eluted from the activity-stained agar strips using SDS-PAGE and staining with silver nitrate. Native-PAGE was performed using the HiTrap Q fraction with the highest activity, and activity staining was performed according to the procedure described in the Materials and Methods. Red arrows in (**a**,**b**) represent the active bands for the enzymes. Red arrows in (**c**,**d**) indicate the predicted positions of Est8L and Est13L, respectively. S, size markers; C, crude extracts; Q, fraction pools from HiTrap Q column chromatography; A, pools from activity staining.

To confirm the size of each cloned enzyme, activity staining was performed using HiTrap Q pools after native-PAGE. In the overlapped-agar strips, the respective active bands of Est8L and Est13L were observed (Figure 4a,b). The region of the gel corresponding to the clear band of the strip was sliced, and then proteins were recovered and analyzed by SDS-PAGE to determine their molecular masses through silver staining. As a result, Est8L and Est13L were observed at the bands corresponding to 33 and 45 kDa, respectively, similar to their expected values (Figure 4c,d).

### 3.3. Determination of Molecular Masses of Native Est8L and Est13L

To determine the molecular masses of native forms of Est8L and Est13L, size exclusion chromatography using a Sephacryl S-200 HR column was performed. The elution volumes of Est8L and Est13L were 61.5 and 47.5 mL, respectively, and their molecular masses were predicted to be 67.2 and 160.0 kDa, respectively. These results indicated that their native forms are a dimer and tetramer, respectively (Table 2).

### 3.4. Properties of Est8L and Est13L

**Figure 5:**
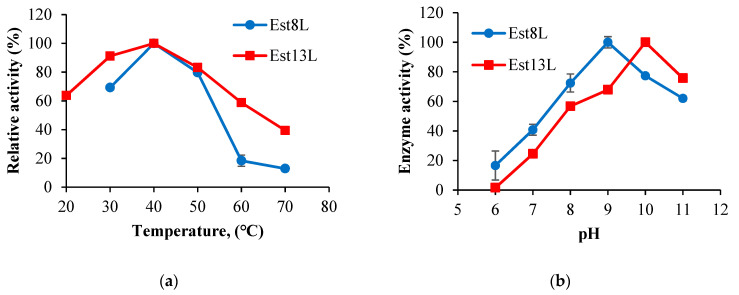
Optimum temperatures (**a**) and optimum pH values (**b**) of Est8L and Est13L.

**Figure 6:**
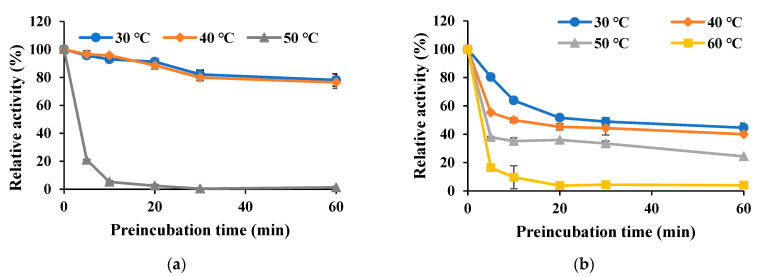
Thermal stabilities of Est8L (**a**) and Est13L (**b**). The enzyme was preincubated at the designated temperatures for the indicated times prior to activity measurements.

Est8L and Est13L were optimally active at 40 °C, and the optimum pH values of Est8L and Est13L were 9.0 and 10.0, respectively, indicating that both are alkaline esterases (Figure 5). Est13L had better thermal stability with a half-life of 3.2 min at 60 °C, whereas Est8L showed a half-life of 3.2 min at 50 °C (Figure 6).

**Figure 7:**
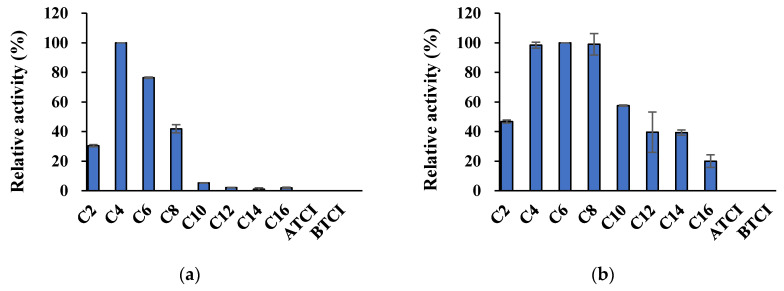
Substrate specificities of Est8L (**a**) and Est13L (**b**). The relative activities were calculated as percentages of the highest activity.

Est8L mostly preferred pNPB (C4), followed by pNP-caproate (C6), pNP-octanoate (C8), and pNP-acetate (C2) with relative activities of 76.5%, 41.8%, and 30.4%, respectively, and showed no significant activities toward other longer substrates (Figure 7). Est13L showed the highest activity for C6, similar to C8 and C4 with relative activities of 99.0% and 98.4%, respectively, and exhibited a moderate activity toward pNP-caprate (C10), C2, pNP-laurate (C12), pNP-myristate (C14), and pNP-palmitate (C16) with relative activities of 57.6%, 46.8%, 39.6%, 39.3%, and 20.0%, respectively, indicating that Est13L had a broad substrate specificity (Figure 7). In contrast, Est8L and Est13L had no significant activity for ATCI and BTCI, which were cholinesterase substrates (Figure 7).

**Figure 8:**
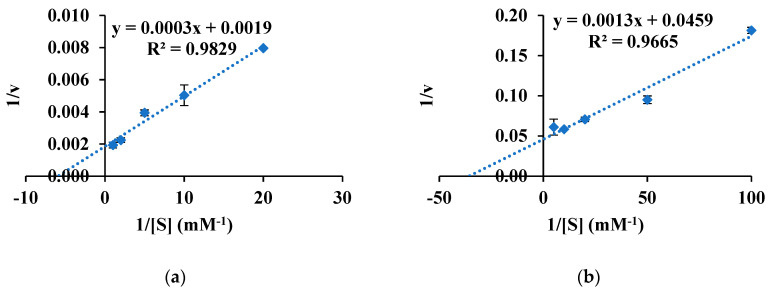
Lineweaver–Burk plots of Est8L (**a**) and Est13L (**b**) for pNPB. Five substrate concentrations were used; they were 0.05, 0.10, 0.20, 0.50, and 1.00 mM for Est8L, and 0.01, 0.02, 0.05, 0.10, and 0.20 mM for Est13L.

In a kinetic study for pNPB (C4) as a substrate, the K_m_ values of Est8L and Est13L were 0.15 ± 0.020 and 0.029 ± 0.0018 mM, respectively, and the V_max_ values were 496.8 ± 64.72 and 22.9 ± 1.51 U/mg, respectively (Figure 8).

**Figure 9:**
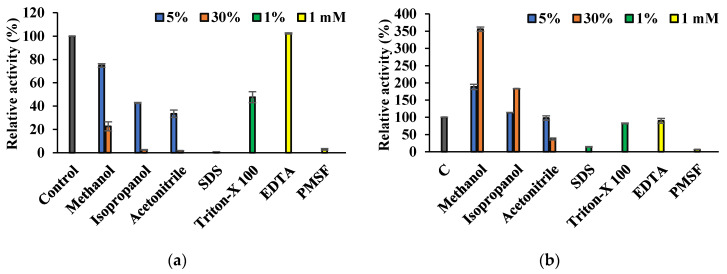
Effects of organic solvents, detergents, and inhibitors on the activities of Est8L (**a**) and Est13L (**b**). The activity was measured using an esterase assay with pNPB as a substrate.

Est8L was significantly inhibited to 22.64%, 2.44%, and 1.76% by 30% methanol, isopropanol, and acetonitrile, respectively, and was moderately inhibited by 47.6% by 1% Triton-X 100 but was almost completely inhibited by 1% SDS (Figure 9a). Est13L was significantly activated to 356.1% by 30% methanol and to 182.9% by 30% isopropanol but was inhibited to 37% by 30% acetonitrile (Figure 9b). Est13L was inhibited to 14.0% by 1% SDS, but its activity remained at 82.4% by 1% Triton-X-100. Est8L and Est13L showed no significant inhibition at 1 mM EDTA; however, both were strongly inhibited by 1 mM PMSF with 2.87% and 6.45% activities, respectively, indicating both are serine esterases (Figure 9).

**Figure 10:**
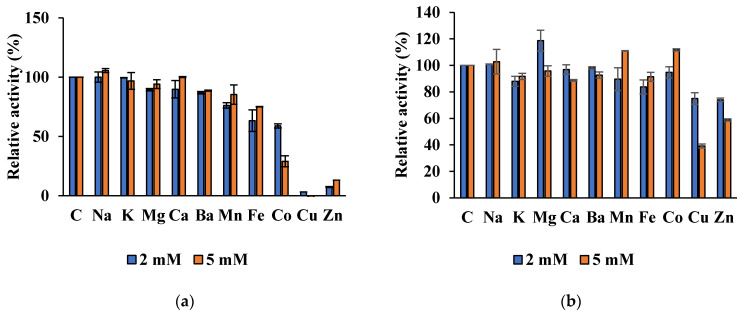
Effects of cations on activities of Es8L (**a**) and Est13L (**b**).

Est8L was significantly inhibited to 0%, 13.2%, and 29.0% at 5 mM Cu^2+^, Zn^2+^, and Cu^2+^, respectively, whereas Est13L was not significantly inhibited by most ions tested, except moderate inhibitions to 39.1% and 58.8% at 5 mM Cu^2+^ and Zn^2+^, respectively (Figure 10).

**Figure 11:**
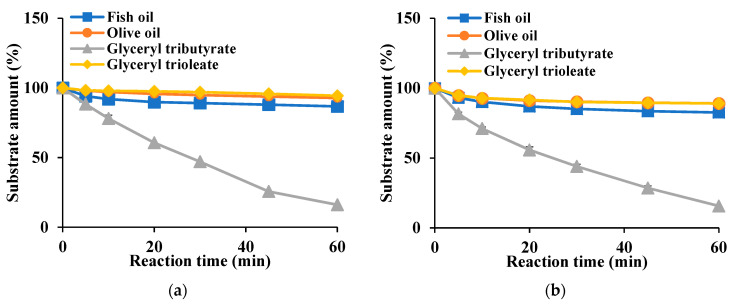
Lipid hydrolysis activities of Est8L (**a**) and Est13L (**b**).

In the hydrolysis of glyceryl esters, Est8L and Est13L preferred glyceryl tributyrate (C4). After hydrolysis for 60 min under the condition described in Materials and Methods, Est8L and Est13L showed similar patterns for hydrolysis of glyceryl triesters and oils; for glyceryl tributyrate, they showed hydrolysis of 16.1% and 15.6%, respectively, and for fish oil, they showed hydrolysis of 86.7% and 82.6%, respectively. Both enzymes showed no significant activities with glyceryl trioleate (C18) and olive oil (Figure 11).

**Figure 12:**
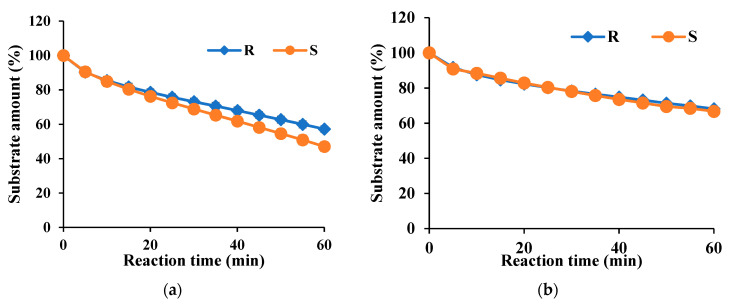
Enantioselectivities of Es8L (**a**) and Est13L (**b**).

In enantioselectivity, Est8L hydrolyzed the *S*-form with 10.1% higher activity than the *R*-form, suggesting preferences for the *S*-form, whereas Est13L showed no selectivity for the enantiomers (Figure 12). In the *t*-test, it was observed that the enantioselectivity for *S*-form was significant compared to *R*-form with a *p*-value < 0.05.

## 4. Discussion

In this study, two novel esterases were characterized. Although the amino acid sequence of Est8L was the same as that of an esterase (MBF6602187) of a metagenome isolated from diarrhea-affected cattle B, the enzymatic properties have not been reported yet. Est8L has low identities (10.7%–38.6%) to other characterized HSLs [33,34,35,36,37]. Est13L was very similar to Est7K, with an identity of 98.5%, and its enzymatic properties were characterized [22]. Est13L was different from Est7K only in six residues, i.e., S36N, K107N, G220E, A236S, S250P, and D265E. However, the properties of Est13L were different from those of Est7K in substrate specificity (C6 vs. C4), enantioselectivity (non-selective vs. *S*-form selective), and effect by Zn^2+^ (inhibited vs. non-inhibited).

In comparison with family IV esterases from similar compost metagenomic sources, Est8L had poor organic solvents stabilities, whereas EstCs1 from a compost metagenomic library was stable at 30% isopropanol, ethanol, acetonitrile, acetone, methanol, dimethyl formamide, and dimethyl sulfoxide [10]. In addition, Est8L was optimally active at pH 10.0, whereas EstCs1 was optimally active at pH 8.0 [10].

In the case of family VIII esterases from similar compost metagenome, Est13L showed similar specific activity EstCs3 from a compost metagenomic library (42.3 vs. 50.2 U/mg). On the other hand, Est13L was stable or activated under organic solvents, whereas EstCs3 was abolished or significantly inactivated by organic solvents such as 30% isopropanol, acetonitrile, ethanol, and methanol [11]. Est13L and EstCs3 showed similar molecular weights; however, their native forms were different (tetramer vs. monomer) [11].

To compare the properties of the Est8L and Est13L with each family, we searched the characterized enzymes at Pubmed (https://pubmed.ncbi.nlm.nih.gov/, accessed on 25 May 2021). The summary of characterization of each enzyme-containing Est8L and Est13L were described (Table 3 and Table 4). Family IV and VIII esterases have been practically interested in the usage of ester synthesis and transesterification reactions, and especially hydrolysis of antibiotics by family VIII [10,11].

Specific activities of Est8L and Est13L at the final purification step were 388.6 and 42.33 U/mg, respectively. Est8L showed a higher specific activity than most family IV esterases such as Est2L (0.22 U/mg) [33], EstKT7 (0.3 U/mg) [38], Est4 (0.64 U/mg) [39], Rv0045c (3.5 U/mg) [40,41], Est3 (3.92 U/mg) [37], Est3K (8.4 U/mg) [18], EstZ (42 U/mg) [42], EstKT4(237.9 U/mg) [38], and E40 (239.2 U/mg) [43,44], except EstKT9 (426.8 U/mg) [38], Est22 (2065 U/mg) [45], AFEST (3000 U/mg) [46,47], and PestE (3910 U/mg) [34,35]. In contrast, Est13L had a moderate specific activity compared to other family VIII esterases, which ranged from 6.7 to 1900 U/mg [48,49,50], and a significantly lower specific activity than Est7K (790.2 U/mg) [22], despite its high identity to Est7K.

The native form of Est8L was observed as a dimer, similar to other HSLs such as PestE [34,35] and Est22 [45] (Table 3). The native form of Est13L was a tetramer, which was unique and unlikely the other family VIII esterases; most family VIII esterases are monomers in their native forms, such as EstC [49], EstBL [51], EstCE1 [52], EstIII [53], and EstCS3 [11]. Est22 was a trimer in its native form [54], and EstA3 required an oligomeric form containing less than six subunits for activation [52] (Table 4).

Est8L and Est13L are alkaline esterases. The optimum pH range of bHSLs is broad, from 5.0 to 9.0. In contrast, the optimum pH range of most family VIII esterases ranges from 7.2 to 10.0, indicating that most family VIII esterases are alkaline esterases. Only a few family VIII esterases have been reported as neutral esterases, the optimum pH of which is 7.0, such as LipBL [55], EstB [56], Lpc53E1 [50], and Lip8 [57] (Table 3 and Table 4).

The optimum temperature (40 °C) of Est8L was similar to that of most bHSLs, which are optimally active at 30–50 °C, except a few cases such as EstE1 (90 °C) [58], PestE (90 °C) [34,35], EST2 (70 °C) [59], and SaHSL (70 °C) [60]. The optimum temperature of family VIII esterases was also distributed from 30 to 50 °C, except LipBL (80 °C) [55] and LipA9 (70 °C) [61] (Table 3 and Table 4). From these results, it could be considered that the optimum temperature (40 °C) for Est8L and Est13L is the average of their family members.

The substrate specificity of bHSLs for pNP esters ranged from C2 to C6, with the most preferred substrates being C2 and C4. Most family VIII esterases prefer C4, likely Est7K [22], which showed the highest identity (98.5%) to Est13L. However, Est13L preferred C6, although the degree was similar to C4 and C8 with broad specificity. Some family VIII esterases prefer C6, such as LipA9 [61], LipBL [55], and DLFae4 [62] (Table 3 and Table 4).

Est8L is sensitive to isopropanol and acetonitrile, similar to Est22 [45], which is inhibited to 40% and 0%, respectively, by 30% isopropanol and acetonitrile. Most reported bHSL family members exhibit poor stabilities for organic solvents such as DMWf18-543, DMwf18-558 [17], Est3 [37], and E69 [63]. In contrast, PestE shows suitable stability under organic solvents, maintaining its activity at ~100% [34,35]. Similar stability was reported at EstCS1 [10] (Table 3). However, Est13L was resistant to or activated in organic solvents under 30% isopropanol or methanol to 183% or 356%, respectively, similar to Est7K [22] but to a greater extent. Similar activations were reported for EstA3 [52], LPC53E1 [50], and EstC [49], which were activated by alcohol solvents. Most family VIII esterases, including Est13L, were inhibited by acetonitrile, but Lpc53E1 was extremely activated to 271.2% under 20% acetonitrile [50] (Table 4).

Est8L showed enantioselectivity for the *S*-form, whereas some bHSLs showed enantioselectivity for the *R*-form, such as EST2 [59] and PestE [34,35] (Table 3). Est13L showed no enantioselectivity, whereas Est7K [22] showed enantioselectivity for the *S*-form, although their sequences were very similar. In family VIII esterases, EstF4k showed enantioselectivity for *R*-form methyl 3-phenylglycidate [64], and EstA3 was highly enantioselective for *S*-form enantiomers [52] (Table 4).

In terms of the ion effects, Est8L showed an inhibition tendency as the metal ion group increased, with moderate inhibition by Co^2+^ and significant inhibitions by Cu^2+^ and Zn^2+^. Similar patterns were reported for other bHSLs such as Est22 [45], SaestA [65], Est4 [39], EstKT7 [35], DMWf18-543, and DMWf18-558 [17] with significant inhibition by Cu^2+^ and Zn^2+^ (Table 3). However, Est13L showed higher stability on metal ions than Est8L, but its pattern was similar, and it was slightly inhibited by Cu^2+^ and Zn^2+^. In family VIII, EstA3 [52] and EstM-N2 [66] were also inhibited by Cu^2+^ and Zn^2+^ (Table 4).

Collectively, Est8L showed a low identity and a higher specific activity than most other family IV esterases reported and different properties in enantioselectivity and sensitivity to the internal proteolytic activity. In contrast, Est13L showed different properties in terms of substrate specificity, specific activity, enantioselectivity, and metal ion inhibition compared to Est7K, although both differed in only six amino acids, similar to the reports that one or several amino acids could cause changes in enzyme properties such as specific activities, substrates specificities, thermostabilities, solvent tolerances, and stereoselectivities in esterases and lipases [67,68,69,70,71].

## 5. Conclusions

In this study, two novel esterase genes *est*8L and *est*13L encoding intracellular Est8L and Est13L, respectively, were isolated from the compost metagenomic library. Est8L and 13 L were revealed to be novel members of family IV esterase (bHSL) and family VIII esterase, respectively. Est8L had similar characteristics to bHSL, such as substrate specificity for C4 and optimum temperature and pH values of 40 °C and 9.0, respectively. Est8L exhibited enantioselectivity for the *S*-form (10.16% higher than the *R*-form). Est13L also had similar characteristics such as molecular weight and optimum temperature and pH. However, Est13L was a tetramer in its native form; unlikely, other family VIII esterases were reported. Est8L was sensitive to isopropanol and acetonitrile, whereas Est13L was activated by 30% isopropanol or methanol. In contrast, Est8L and Est13L were inhibited by Cu^2+^ and Zn^2+^ with similar patterns, but Est13L was more stable than Est8L. In conclusion, Est8L had higher specific activity than most of other HSLs and enantioselectivity for the *S*-form; however, it was sensitive to organic solvents; Est13L had higher stability against ions, organic solvents such as alcoholic solvents (isopropanol and methanol), unusual tetrameric form in family VIII esterases, and thermal stress. These results suggested that Est8L and Est13L can be used for chemical reactions with enantioselectivity and for detergent/chemical reactions under alkaline conditions, respectively.

## Figures and Tables

**Table 1:** Purification of Est8L and Est13L using chromatographic procedures.

Enzyme.	Preparation	Specific Activity (U/mg)	Purification (Fold)	Yield (%)
Est8L	Crude extract	32.6	1.0	100
	HiTrap Q	268.6	8.2	61.8
	MMC	388.6	11.9	12.1
Est13L	Crude extract	1.05	1.00	100
	HiTrap Q	10.79	10.3	68.6
	HIC	42.33	40.3	43.2

**Table 2:** Molecular masses of native Est8L and Est13L determined by Sephacryl S-200 HR.

	Fraction Volume (mL)	Molecular Mass (kDa)	log Mw
β-amylase	46	200.0	2.30
BSA	58	66.4	1.82
Trypsinogen	78	24.0	1.38
Est8L	61.5	67.2 ± 2.9	1.83
Est13L	47.5	160.0 ± 7.3	2.20

The means ± SEs were calculated via duplicate experiments.

**Table 3:** Comparison of Est8L and other bacterial hormone-sensitive lipases (bHSLs).

Protein	Accession	Source	Homology (%)	AA	MW (kDa)	Native Form *	Opt Temp. (°C)	Opt. pH	Preferred pNP Esters	Organic Solvent Effects (%) **			Enantio-Selectivity	Ion Effect ***	Ref.
										IPA	MeOH	ACN			
Est8L	MZ484407	Uncultured bacterium		314	33.2	Di	40	9.0	C4	42.68 ^a^	74.8 ^a^	33.4 ^a^	*S*	(-)Cu^2+^, (-)Zn^2+^, (-)Co^2+^-	This study
EstCS1	AEQ63714	Uncultured bacterium	31.58	309	34.5		50	8.0	C3, C6	115.8 ^a^	90.8 ^a^	101.1 ^a^		(-)Cu^2+^, (-)Zn^2+^, (-) Co^2+^, (+)Ca^2+^	[10]
DMWf18-543	AUF80930	Uncultured bacterium	22.74	302	32.3		40	7.0	C4	0 ^c^	0 ^c^	0 ^c^		(-)Cu^2+^, (-)Zn^2+^, (-)Ni^2+^, (-)Co^2+^	[17]
DMWf18-558	AUF80945	Uncultured bacterium	22.81	302	32.1		40	7.0	C2	0 ^c^	0 ^c^	0 ^c^		(-)Cu^2+^, (-)Ni^2+^, (-)Co^2+^, (-)Zn^2+^,	
Est3K	AKG92633	Uncultured bacterium	21.25	299	32.4		50	9.0	C4	87.5 ^a^	86.9 ^a^	47.7 ^a^		(-)Cu^2+^	[18]
Est2L ^#^	MT989338	Uncultured bacterium	10.69	839	92.5	Di	60	10.0	C2	15.7 ^a^	92.7 ^a^	47.7 ^a^	*S/R*	(+)Mg^2+^, (+)Mn^2+^, (+)Fe^2+^, (-)Cu^2+^	[33]
PestE	BAC06606	*Pyrobaculum calidifontis*	34.27	313	34.4	Di	90	7.0	C6	110 ^b^	109 ^b^	117 ^b^	*R*		[34,35]
Est25	AAY45707	*Uncultured bacterium*	22.68	362	38.3	Di	25	7.0	C4						[36]
Est3	ALP75907	*Parvibaculum* sp.	38.56	312	32.8		41	6.0	C5	38.2 ^b^	47.7 ^b^			(+)Mn^2+^, (+)Li^2+^	[37]
EstKT4	ADH59412	Uncultured bacterium	19.34	352	38.2		40	8.5	C5					(-)Mn^2+^, (-)Zn^2+^, (-)Ni^2+^, (+)K^+^	[38]
EstKT7	ADH59413	Uncultured bacterium	20.76	316	34		35	8.0	C5					(-)Cu^2+^, (-)Zn^2+^, (-)Ni^2+^, (+)Na^+^	
EstKT9	ADH59414	Uncultured bacterium	16.53	372	40.8		45	8.5	C5					(-)Zn^2+^	
Est4	CCI69497	*Rhodococus* sp.	20.97	313	33.2		30	7.0	C4					(-)Cu^2+^, (-)Zn^2+^, (-)Fe^2+^, (-)Ag^2+^,	[39]
Rv0045c	I6XU97	*Mycobacterium tuberculosis*	10.79	298	32.1		39	8.0	C6						[40,41]
EstZ	AAM16269	*Pseudomonas putida*	30.46	318	34.3		40	7.5	C2						[42]
E40	AKF17659	*Uncultured bacterium*	20.89	297	32.1	Tet	45	8.0	C4					(+)Na^+^	[43,44]
Est22	AFB82695	Uncultured bacterium	22.06	344	36.7	Di	40	7.5	C2	40 ^c^	120 ^c^	0 ^c^		(-)Zn^2+^, (-)Cu^2+^	[45]
AFEST	WP_010879212	*Archaeoglobus fulgidus*	32.92	311	35.5	Mono	80	7.1	C6						[46,47]
EstE1	AAW62260	Uncultured archaeon	31.25	311	33.8	Di	95	6.0	C6						[58]
EST2	QGT40748	*Alicyclobacillus acidocaldarius*	31.25	311	34.4	Mono	70	7.1	C6				*R*		[59]
SaHSL	AEP27067	*Salinisphaera* sp.	36.45	316	34.4		70		C2	75^a^				(+)Na^+^	[60]
E69	AUD08548	*Erythrobacter seohaensis*	17.87	274	29.5		60	10.5	C4	0 ^c^	28.8 ^c^	35.0 ^c^		(+)Na^2+^	[63]
SAestA	AIY29984	*Salinispora arenicola*	29.00	324	33.8		25	9.0	C4		113 ^d^			(-)Hg^2+^, (-)Cu^2+^, (-)Zn^2+^	[65]

* Mono, monomer; Di, dimer; Tet, tetramer. ** IPA, isopropanol; MeOH, methanol; ACN, acetonitrile. Organic solvent stability at concentration of ^a^ 30%, ^b^ 50%, ^c^ 15%, and ^d^ 25%, *** (+), activated; (-), inhibited. ^#^ Because Est2L is a fusion-type protein, esterase domain (421 AA) was used for the identity analysis.

**Table 4:** Comparison of Est13L and other family VIII esterases.

Protein	Accession	Source	Homology (%)	AA	MW (kDa)	Native Form *	Opt Temp. (°C)	Opt. pH	Preferred pNP Esters	Organic Solvent Effects (%) **			Enantio-Selectivity	Ion Effect ***	Ref.
										IPA	MeOH	ACN			
Est13L	MZ484408	Uncultured bacterium		411	44.9	Tet	40	10.0	C6	182.9 ^a^	356.1 ^a^	37.0 ^a^	*S/R*	(-)Cu^2+^, (-)Zn^2+^	This study
EstCS3	ARE60547	Uncultured bacterium	14.0	409	44.5	Mono	55	8.0	C4	0.5 ^a^	44.5 ^a^	2.2 ^a^			[11]
Est7K	AJN91095	Uncultured bacterium	98.5	411	45.0		40	10.0	C4	106.5 ^a^	208.5 ^a^	33.7 ^a^	*S*	(-)Cu^2+^	[22]
PBS-2	AHL66978	*Paenibacillus* sp. PBS-2	30.0	377	42.2		30	9.0	C4	104 ^a^	102 ^a^	62 ^a^		(-)Hg^2+^, (-)Cu^2+^	[48]
EstC	ACH88047	Uncultured bacterium	39.3	427	46.3	Mono	40		C4	~100 ^a^	~600 ^a^				[49]
Lpc53E1	AFM09717	Uncultured bacterium	31.4	387	41.9		40	7.0	C4	176.4 ^b^	220.5 ^b^	271.2 ^b^		(-)Ba^2+^, (-)Mg^2+^, (-)Rb^2+^, (+)Na^+^	[50]
EstBL	AAX78516	*Burkholderia cepacia*	31.0	398	42.0	Mono			C2						[51]
EstA3	AAZ48934	Uncultured bacterium	57.6	396	43.3	Hexa	50	9.0	C4	117 ^a^	130 ^a^	87 ^a^	*S*	(-)Ca^2+^, (-)Cu^2+^, (-)Mg^2+^, (+)Co^2+^	[52]
EstCE1	AAY90134	Uncultured bacterium	25.9	378	41.3	Mono	47	10.0	C4	23 ^a^	0 ^a^	0 ^a^	*S*	(-)Cu^2+^	
EstIII	AAC60471	*Pseudomonas fluorescens* SIK WI	35.1	382	41.8	Mono	50	9.5	C2						[53]
Est22	AGT17593	Uncultured bacterium	34.7	423	47.0	Tri	30	8.0	C4						[54]
LipBL	KPP97659	*Marinobacter* sp. HL-58	49.1	404	45.4		80	7.0	C6	98.4 ^a^	120.5 ^a^	27.7 ^a^	*S*		[55]
EstB	AAF59826	*Burkholderia gladioli*	31.0	392	41.7		43	7.0	C4					(-)Na^+^, (-)Hg^2+^	[56]
Lip8	BAD69792	*Pseudomonas aeruginosa*	30.7	391	42.1		30	7.0	C2		~0 ^b^				[57]
LipA9	AYH52116	*Marinobacter lipolyticus*	47.1	404	45.2		70	8.0	C6	90~100 ^a^	90~100 ^a^	60~80 ^a^			[61]
DLFae4	AZK36008	Uncultured microorganism	28.0	338	37.2		50	8.6	C6	20~30 ^a^	~15 ^a^			(-)Cu^2+^, (-)Co^2+^	[62]
EstF4k	AEH57832	Uncultured bacterium	57.5	396	43.1		50	8.0	C3, C4		175.8 ^a^	16.5 ^a^	*S*	(-)Hg^2+^, (-)Ag^2+^	[64]
EstM-N2	AEA07655	Uncultured bacterium	46.0	407	45.6		30	9.0	C4					(-)Fe^2+^, (-)Cu^2+^	[66]

* Mono, monomer; Tri, trimer; Tet, tetramer; Hexa, hexamer. ** IPA, isopropanol; MeOH, methanol; ACN, acetonitrile. Organic solvents effect at ^a^ 30%, ^b^ 20%. *** (+), activated; (-), inhibited.

## Data Availability

The data presented in this study are available on request from the corresponding author.

## Supplementary Materials

The following are available online at https://www.mdpi.com/article/10.3390/microorganisms9081614/s1.
