# Supplementary material for: Molecular Characterization of Novel Family IV and VIII Esterases from a Compost Metagenomic Library
Source: Microorganisms. 2021 Jul 29;9(8):1614. doi: 10.3390/microorganisms9081614 (PMC8399190; doi:10.3390/microorganisms9081614)
Supplement: Supplementary file 1 [file microorganisms-09-01614-s001.zip › microorganisms-1312052-supplementary.pdf]

Supplementary Materials

Molecular characterization of novel family IV and VIII esterases from a compost metagenomic library

Jong Eun Park, Geum Seok Jeong, Hyun Woo Lee, Hoon Kim*

Department of Pharmacy, and Research Institute of Life Pharmaceutical Sciences, Sunchon National University, Suncheon 57922, Republic of Korea; 1200113@s.scnu.ac.kr (J.E.P); 1200058@s.scnu.ac.kr (G.S.J); hwlee@rophibio.com (H.W.L)
*Correspondence: hoon@sunchon.ac.kr; Tel.: +82617503751

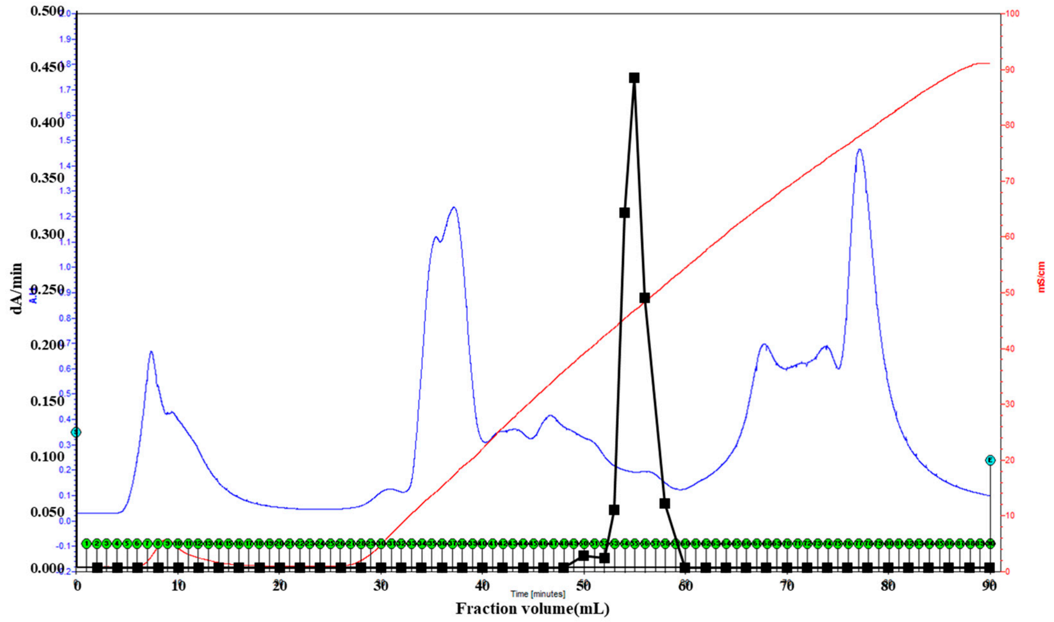

(**a**)

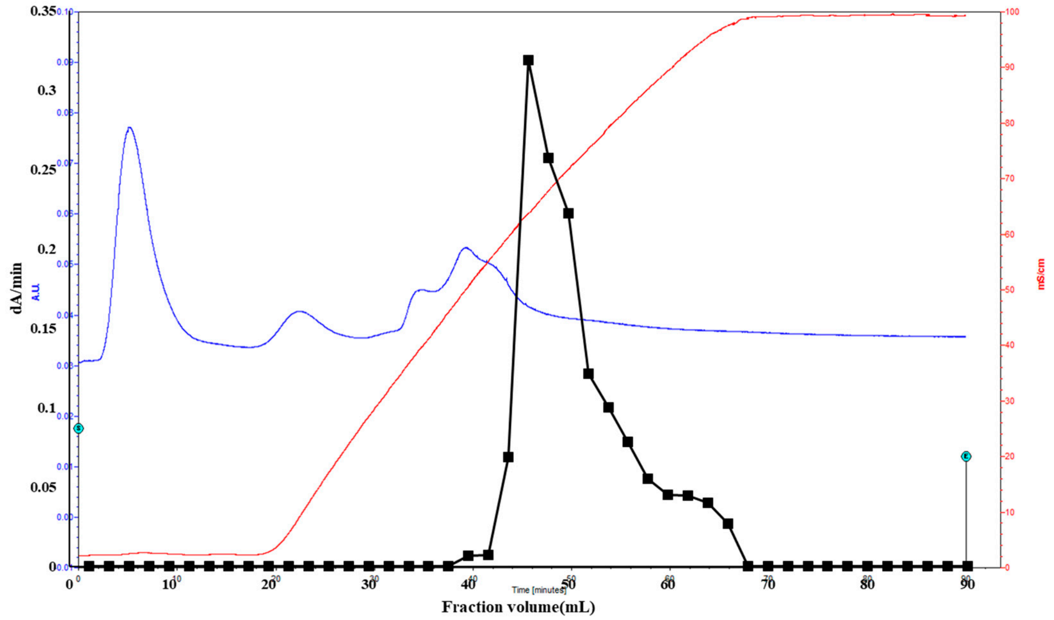

(**b**)

Figure S1. HiTrap Q (**a**) and HiTrap capto MMC (b) chromatogram of Est8L recorded by BioLogic LP system (Bio-Rad, California, USA). The activities of fractions were marked as black line and points. The blue and red lines represent UV absorbance (A.U.) at 280 nm and conductivity (mS/cm), respectively.

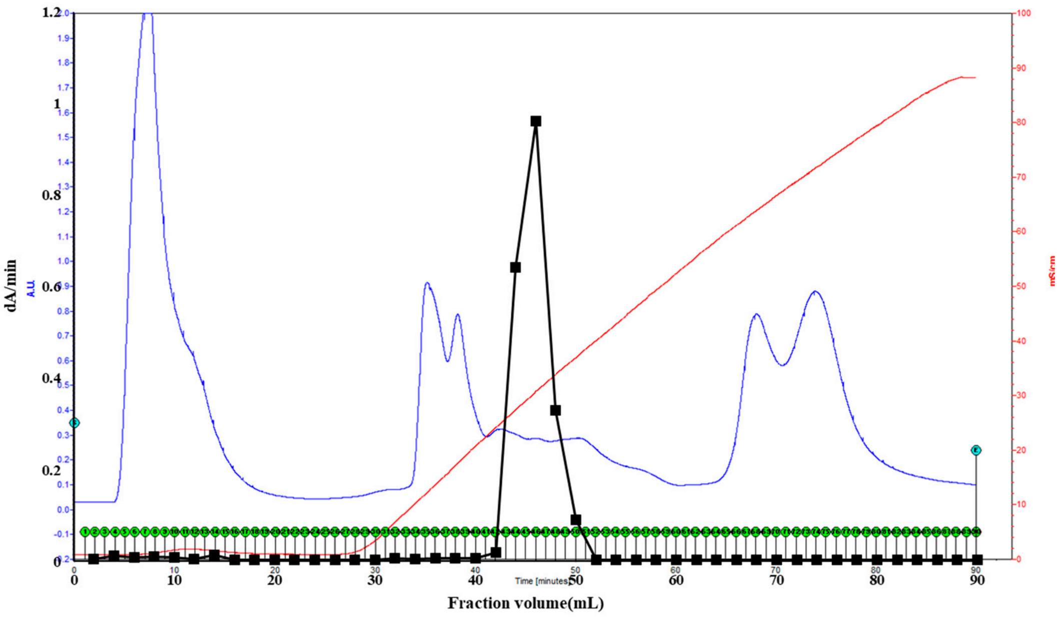

(**a**)

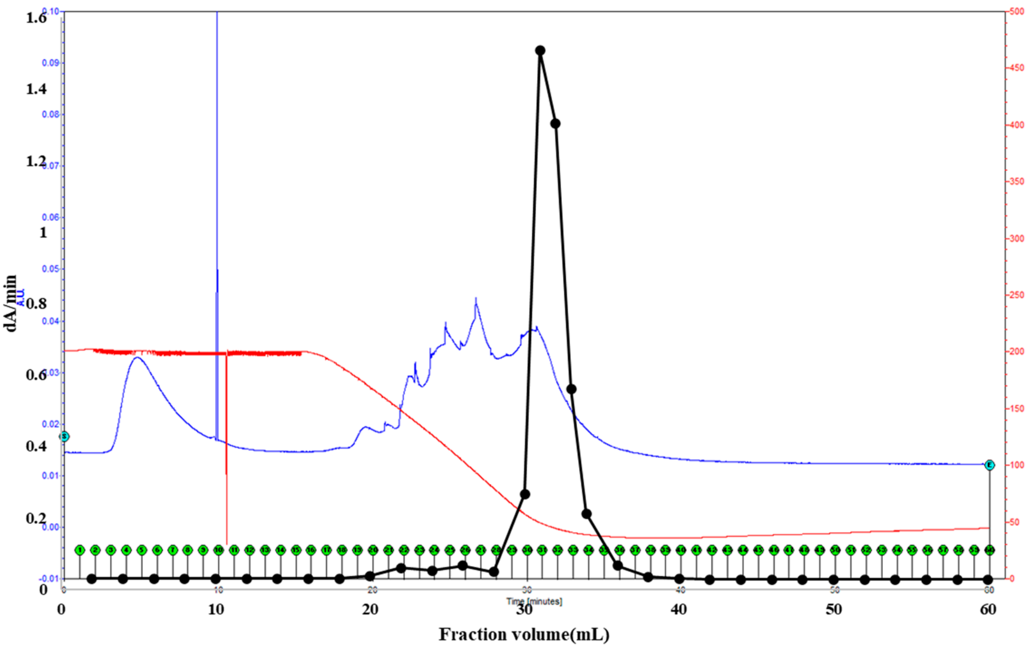

(**b**)

Figure S2. HiTrap Q (**a**) and *t*-Butyl HIC (**b**) chromatogram of Est13L recorded by BioLogic LP system (Bio-Rad, California, USA). The activities of fractions were marked as black line and points. The blue and red lines represent UV absorbance (A.U.) at 280 nm and conductivity (mS/cm), respectively.
